# Rescue of Recombinant Adenoviruses by CRISPR/Cas-Mediated *in vivo* Terminal Resolution

**DOI:** 10.3389/fmicb.2022.854690

**Published:** 2022-03-18

**Authors:** André Riedl, Julian Fischer, Hans-Gerhard Burgert, Zsolt Ruzsics

**Affiliations:** Faculty of Medicine, University Medical Center Freiburg, Institute of Virology, University of Freiburg, Freiburg, Germany

**Keywords:** recombinant adenoviruses, CRISPR/Cas9, adenovirus reconstitution, bacmid vector, human adenovirus species C, human adenovirus species E

## Abstract

Recombinant adenovirus (rAd) vectors represent one of the most frequently used vehicles for gene transfer applications *in vitro* and *in vivo*. rAd genomes are constructed in *Escherichia coli* where their genomes can be maintained, propagated, and modified in form of circular plasmids or bacterial artificial chromosomes. Although the rescue of rAds from their circular plasmid or bacmid forms is well established, it works with relatively low primary efficiency, preventing this technology for library applications. To overcome this barrier, we tested a novel strategy for the reconstitution of rAds that utilizes the CRISPR/Cas-machinery to cleave the circular rAd genomes in close proximity to their inverted terminal repeats (ITRs) within the producer cells upon transfection. This CRISPR/Cas-mediated *in vivo* terminal resolution allowed efficient rescue of vectors derived from different human adenovirus (HAdV) species. By this means, it was not only possible to increase the efficiency of virus rescue by about 50-fold, but the presented methodology appeared also remarkably simpler and faster than traditional rAd reconstitution methods.

## Introduction

Recombinant adenoviruses (rAds) are one of the predominantly used vectors for both recombinant vaccines and gene therapy [see (Gao et al., 2019; Bos et al., 2020; van Doremalen et al., 2020; Logunov et al., 2021) for some recent milestones]. Usually, rAds are constructed in *Escherichia coli* where their genome is maintained as circular plasmid (Ghosh-Choudhury et al., 1986) or bacmid (Ruzsics et al., 2006). Since adenoviruses replicate without a circular intermediate (Russell, 2000), for the reconstitution of rAds, conventionally, the circular constructs are processed by restriction endonuclease treatment prior to or upon transfection into producer cells (Gao et al., 2003, 2019) or circular and linear genome parts are recombined in the cells (Hardy et al., 1997). The rescue of rAds directly from circular DNA may be facilitated by fusion of their inverted terminal repeats (ITRs) (Graham, 1984). However, even though it was shown to be as efficient as transfection of linear adenovirus DNA, the ITR fusion did not allow direct removal of the bacterial vector sequences, preventing rescue of wild-type viruses or decreased vector capacity. Since transfection of linear DNA is less efficient than introduction of circular DNA (Chancham and Hughes, 2001), it has been shown that rAd vector cleavage in cells by co-transfection of restriction endonuclease expression vectors is more efficient than virus rescue by *in vitro* linearized DNA (Gao et al., 2003). Although this improved the primary efficiency of rAd rescue by about fivefold (Stanton et al., 2008), *in vitro* enzymatic linearization is still the standard methodology for rAds rescue, since most applications with standard rAd-based vectors require only reconstitution of a single recombinant at a time and this approach has an impressive simplicity compared to other techniques. However, the establishment of new rAd vectors based on other HAdV species and types, or based on animal adenoviruses, and the *in vitro* linearization technique may have methodical limitations at the level of virus rescue (Ibanes and Kremer, 2013). Furthermore, the relative inefficiency of primary virus rescue using any of the above-mentioned methodologies prevented direct library applications based on rAds, which are readily available for other viral vector platforms, such as recombinant lenti- or adeno-associated viruses.

To overcome these barriers, we attempted to improve the basic methodology of rAd rescue. Commonly used restriction endonucleases cannot release the ITRs of the rAds precisely, since their recognition sequence including their cleavage sites must be introduced outside of the ITR ends. This way, restriction endonuclease cleavages always leave double-stranded DNA overhangs, with or without additional single-stranded overhangs on the genome ends artificially extending the ITRs. This may inhibit or delay the recovery of protein priming for adenoviral genome replication, which is directed to the exact termini (Kenny and Hurwitz, 1988). In contrast, in experimental settings, Cas9 nuclease can introduce DNA double-strand breaks on almost any freely chosen DNA target when directed by a specific single-guide RNA [sgRNA, reviewed in Wang et al. (2016)] upon the presence of the protospacer adjacent motif (PAM). Due to this nucleic acid guide, Cas9 can be directed to induce DNA cleavage exactly and with great specificity, which is unique among nucleases. Therefore, this may also provide a unique possibility to realize the ends of rAd exactly upon vector rescue.

Here, we show that application of the so-called CRISPR/Cas-mediated *in vivo* terminal resolution (CTR) to release the rAd genome ends upon transfection of circular DNA resulted in a more efficient reconstitution of rAds, both with respect to an increase in primary plaque formation and shortening the time of rescue in some cases. We could show that, similar to the other nuclease-based techniques, using a generally applicable external target sequence allowed efficient rescue of different rAd species. Moreover, we could demonstrate that exact cleavage at the ends of the ITRs of circular rAds is possible, when specifically designed sgRNAs are used, and this exact cleavage results in even more efficient vector rescue. By using CTR directed to exact cleavage at the ITRs of rAd-based HAdV-5-bacmids, we could increase the primary rescue efficiency by about 50-fold, which seems to make basic library applications for this vector platform feasible.

## Materials and Methods

### Cells, Viruses, and Bacteria

293A cells are low-passage 293 cells (ATCC CRL1573) (Invitrogen, Carlsbad, CA, United States), A549 (ATCC CCL-185) are human lung carcinoma, and SKOV-3 (ATCC HTB-77) are human ovary cells. All cell lines were cultured in Dulbecco’s modified Eagle’s medium (DMEM) supplemented with fetal calf serum (FCS, 10% v/v, Sigma-Aldrich, St. Louis, MO, United States) and penicillin–streptomycin (100 U/ml, Gibco, Carlsbad, CA, United States). To generate 293A-based cell lines with stable expression of codon-optimized SpCas9 (293A-Cas9-B2 and 293A-Cas9-b5), we co-transfected low-passage 293 cells (herein called 293A) with linearized pSG5-Cas9F (described below) and *Pvu*I-treated pGC-neo (Korner et al., 1992) by Gene Pulser electroporation using the 25.5 ms square wave protocol at 310 V and infinite resistance. The cells were selected by 500 μg/ml G418 (Formedium, Norfolk, United Kingdom). Single clones were picked, expanded, and analyzed by flow cytometry (FACS) of Cas9-Flag, detecting the Flag-tag by anti-Flag-M2 (Sigma-Aldrich, St. Louis, MO, United States). In addition, all clones were tested for their permissiveness of rAd rescue by co-transfecting pBWH-C5-mChe and the sgRNA expressing helper plasmid pAR-gRNA-Ex (see below) for the evaluation of the rescue proficiency of each picked clone. Two clones (B2 and b5, see Figure 4A) were selected for use as adenovirus E1 complementing, Cas9-expressing cell lines in this study (referred as 293A-Cas9-B2 and 293A-Cas9-b5).

**FIGURE 1:**
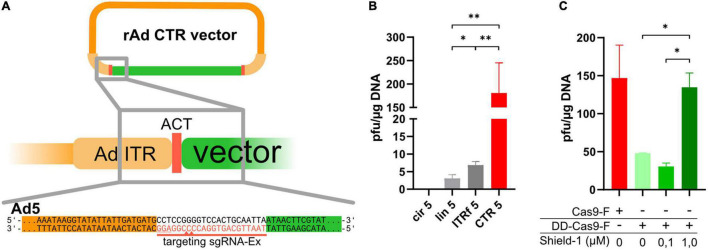
CRISPR-Cas-mediated cleavage in cells yielded efficient rescue of recombinant adenoviruses based on HAdV-5. **(A)** The ITRs on an existing rAd-plasmid were extended by artificial CRISPR/Cas9 target sequences (*ACT sequences*, red), which were targeted by sgRNA-Ex (red line, PAM is underlined) inducing double-strand breaks 6–7 bp outside of the ITRs (indicated by the red triangles on the sgRNA targeting strand only). **(B)** Reconstitution efficiency of rAds in 293A cells after transfection of circular pBWH-C5-mChe (an ACT-flanked Ad5-bacmid) alone (cir5), a linearized rAd5 bacmid (lin5), a circular rAd5 bacmid carrying fusion ITRs (ITRf 5) or co-transfection of circular pBWH-C5-mChe with pAR-gRNA-Cas9F-Amp, a Cas9, and gRNA-Ex expressing helper plasmid for CRISPR/Cas-mediated terminal resolution (CTR 5). The appearing plaques were counted after seeding the transfectants into multiwell plates by observing the plates for 14 days after transfection. Statistical significance was determined using Welch ANOVA tests. **(C)** The rAd reconstitution efficiencies (as foci/μg DNA) in 293A cells of pBWH-C5-mChe-DD-Cas9, expressing conditional Cas9 (green bars) with co-expression of sgRNA-Ex, in the presence of stabilizer Shield-1 at the indicated concentrations are compared to the efficiency of CTR using Cas9F as for CTR 5 in **(B)** (red bar). The primary rescue efficiencies were obtained as in **(B)**. Significance was calculated using ordinary one-way ANOVA. *p < 0.05 and **p < 0.01.

**FIGURE 2:**
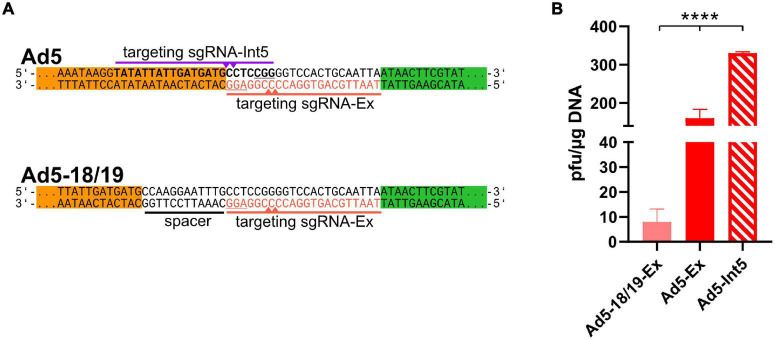
ITR-near CRISPR-Cas-mediated cleavage increased the efficient of recombinant adenoviruses rescue. **(A)** Another sgRNA (sgRNA-Int5, purple, PAM underlined) targeting the ITRs (bold) can induce Cas9-mediated cleavage at the ITRs (purple triangles). To check the impact of cleavage distance, the ITRs were extended by CRISPR/Cas9 target sequences (*ACT*, red) using a 12-bp long spacer (black underlined), which was targeted by sgRNA-Ex (red, PAM is underlined) inducing double-strand breaks (red triangles) 18–19 bp upstream of the ITRs. **(B)** rAd reconstitution efficiencies were compared after co-transfection of 293A cells with pBWH-C5-mChe and pSG5-Cas9F in the presence of either sgRNA-Int5 (Ad5-Int5) or sgRNA-Ex (Ad5-Ex), and with pBWH18/19-C5-mChe in the presence of sgRNA-Ex (Ad518/19-Ex). The primary rescue efficiencies were obtained as in Figure 1B. Significance was calculated using Welch ANOVA test. ****p < 0.0001.

**FIGURE 3:**
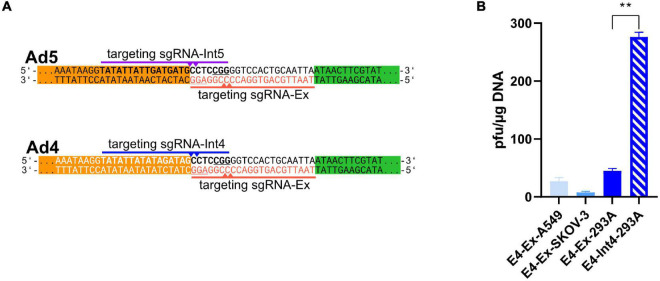
ITR-near CTR induced also efficient rescue of recombinant adenoviruses based on HAdV-4. **(A)** Similarly to the HAdV-5-based constructs (upper panel), a recombinant HAdV-4 based bacmid was constructed (lower panel), which was flanked with ACT sequences (red, PAM is underlined) at both ITRs (here, the right ITR is shown, white letters). This sequence can be targeted by the universal sgRNA-Ex, like for the HAdV-5 based constructs (upper panel). Here also another sgRNA (sgRNA-Int4, blue, PAM is underlined) targeting the HAdV-4 ITRs (bold) can induce Cas9-mediated cleavage at the ITRs (blue triangles). **(B)** The rAd reconstitution efficiencies were determined after co-transfection of A549, SKOV-3, and 293A cells with pBWH-E4-ΔE3 with either pAR-gRNA-Cas9F-Amp expressing sgRNA-Ex (E4-Ex) or with pAR-Int4-Cas9F-Amp (E4-Int4, for 293A cells only). The primary rescue efficiencies were obtained as in Figure 1B. A549-Ex and SKOV-3-Ex were tested twice in technical duplicates, and error bars represent spread of results. E4-Ex and E4-Int4 were done three times in technical duplicates, and error bars represent standard deviation. Significance was calculated using unpaired *t*-test comparing E4-Ex and E4-Int4. **p < 0.01.

**FIGURE 4:**
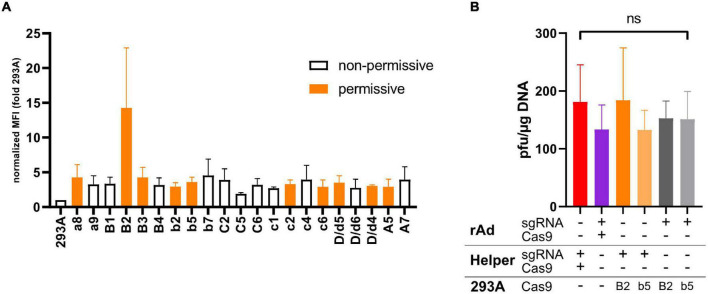
**(A)** Shown is the fold increase in the mean fluorescent intensity (MFI) in Flag-tag-specific FACS measurements of different 293A-Cas9 clones expressing Flag-tagged Cas9 compared to the parental 293A signal. Data shown represent the mean of two independent experiments, with error bars indicating the range of measurements. All presented clones were also checked for CTR competency of rAd rescue by co-transfecting of pBWH-C5-mChe and pAR-gRNA-Ex. Clones that appeared to be permissive for rAd rescue based on two experiments are indicated by orange bars, while the white bars indicate clones, which appeared to be non-permissive in at least one experiment. **(B)** Comparison of different approaches for supplying necessary components for CRISPR/Cas9-mediated CTR. Co-transfection of helper plasmid(s) (Helper) expressing sgRNA and Cas9 protein with the construct carrying the ACT flanked rAd bacmids (rAd, red bar, this approach was applied for most of the experiments described in the manuscript) was compared to combination of all CTR components in one construct coding for the rAd genome and all necessary CRISPR/Cas9-components in the vector backbone (purple bar); the Cas9 is delivered by constitutive expression in the cell line B2 or b5 [see **(A)**] used to rescue the rAd, while a bacmid coding for a rAd genome is co-transfected together with a sgRNA-expressing plasmid as in panel **(A)** (dark and light orange bars); and the same as in previously, but sgRNA is expressed from the same construct that carries the rAd (dark and light gray bars). The primary rescue efficiencies were obtained as in Figure 1B. Significance was calculated using Welch’s ANOVA test.

The HAdV-5-derived first-generation vectors were based on published constructs (Ruzsics et al., 2014). The HAdV-4 (strain RI-67) was obtained from the ATCC (VR-1572). The primary stock was amplified on 293A cells and was used for DNA isolation after three tissue culture passages.

The *E. coli* strains NEB 10-beta [genotype: Δ(*ara-leu*) 7697 araD139 fhuA ΔlacX74 galK16 galE15 e14- Φ80dlacZΔM15 recA1 relA1 endA1 nupG rpsL (StrR) rph spoT1 Δ(mrr-hsdRMS-mcrBC)] and NEB 5-alpha [genotype: fhuA2 Δ(argF-lacZ) U169 phoA glnV44 Φ80 Δ(lacZ)M15 gyrA96 recA1 relA1 endA1 thi-1 hsdR17] were purchased from New England Biolabs (Frankfurt, Germany). *E. coli* Pir-1 (Invitrogen, Carlsbad, CA, United States) [genotype: F- Δlac169 rpoS(Am) robA1 creC510 hsdR514 endA recA1 uidA(Δ*Mlu*I)::pir-116] was used to maintain plasmids with ori6Kγ-based origin of replication (ori).

### Plasmids

pO6-A5-mChe was constructed by inserting the mCherry ORF from pmCherry-C1 (Takara, Kusatsu, Japan) into pO6A5-CMV (Ruzsics et al., 2006). pO6-A5-WH-CMV-mChe was constructed by inserting an experimentally proven Artificial CRISPR-Cas-Target site [TAATTGCAGTGGACCCCGGAGG (Yuen et al., 2017), referred here as *ACT* sequence] directly adjacent to the left ITR of the HAdV-5 present in this vector (Ruzsics et al., 2014). Similarly, pO6-A5-WH18/19-mChe was constructed by inserting the same *ACT* sequence, but with a 12-base pair spacer (CAAATTCCTTGG) between the ACT sequence and the ITR.

pO6-A5F-CMV-GFP was a modified version of pO6A5-CMV (Ruzsics et al., 2014), which carried the ITR-fusion of pFG40 (Graham, 1984) instead of the wild-type HAdV-C5 ITR.

The expression cassette coding for the destabilized, human codon-optimized Cas9 fusion protein (DD-Cas9) was constructed on the basis of pDD-Cas9 [Addgene Plasmid #90086, a kind gift from Raffaella Sordella (Senturk et al., 2017)] by inserting a glutamine codon instead of the first methionine codon of the Cas9 coding sequence. The constitutively active C-terminally Flag-tagged Cas9 cassette (Cas9F) was also derived from pDD-Cas9 by deleting its DD domain. The sgRNA-Ex expression cassette was synthetized by fusing the U6-promoter sequence (GenBank accession no. JN255693.1) to the sgRNA scaffold containing an sgRNA targeting-site with low off-target activity, described by Yuen et al. (2017). The pH-gRNA-iGFP, the pH-gRNA-DDD-Cas9-iGFP, and the pH-gRNA-Cas9F-iGFP plasmids were constructed by inserting sgRNA-Ex alone or together with one of the above-described versions of the Cas9 expression cassette into vector pH-iGFP (GenBank accession no. MT219956) carrying a conditional bacterial ori (Hashimoto-Gotoh et al., 1981). These constructs were used to insert the CRISPR/Cas-components into the rAd genome containing bacterial artificial chromosomes (BACs) at their rox site (see below). pSG5-Cas9F, the expression vector, which was used to generate stable Cas9-expressing cell lines, was constructed by inserting the Flag-tagged Cas9 ORF from pH-gRNA-Cas9F-iGFP into the pSG5 expression vector (Agilent Technologies, Inc., Santa Clara, CA, United States). The high-copy plasmid pAR-gRNA-Cas9F-Amp coding for the gRNA-Ex and the Cas9 expression cassettes from pH-gRNA-Cas9F-iGFP was constructed by inserting the respective CRISPR/Cas components into pcDNA3.1 (Thermo Fisher Scientific, Waltham, MA, United States). The pAR-gRNA-Ex, coding for the sgRNA-Ex expression cassette alone, was constructed by amplifying the respective part from pAR-gRNA-Cas9F-Amp by PCR and re-ligation. The pAR-gRNA-Int5 coding for the exactly cleaving sgRNAs specific for HAdV-5 ITRs (Int5) was constructed by replacing the external targeting sequence of pAR-gRNA-Ex with the corresponding internal targeting sequences (TATATTATTAGATAGCCTC). The pAR-Int4-Cas9F-Amp coding for exactly cleaving sgRNAs specific for HAdV-4 and Cas9F was constructed by replacing the external targeting sequence of pAR-gRNA-Cas9F-Amp with the corresponding internal targeting sequences for Int4 (TATATTATATAGATAGCCTC).

The newly constructed plasmids described above were verified by Sanger sequencing, and the sequences of the functionally relevant final constructs were submitted to GenBank. The basic features of the plasmids generated in this study including their GenBank accession numbers are summarized in Supplementary Table 1.

### BACs Carrying Recombinant Adenovirus Genomes

The bacterial rAd constructs were generated by either modifying published rAd constructs to make them compatible to the new rescue technique presented here or constructed for this study directly from purified adenovirus DNA. To modify existing constructs, we inserted specifically designed artificial CRISPR-Cas target sequences (*ACT sequences*) into the rAd genomic constructs adjacent to their ITRs and optionally one or more of the above-described components of the CRISPR-Cas system. To modify genomic constructs, we used either recombineering (Datsenko and Wanner, 2000) or single-step site-specific recombination (3SR) (Riedl et al., 2020).

The pBWH-C5-mChe, representing a HAdV-C5-based first-generation vector (ΔE1 and ΔE3) was constructed in two steps. First, one copy of *ACT sequence* was introduced into pBA5-FRT adjacent to its right ITR, by a two-step recombineering using a synthetic linear DNA fragment as donors, which at the same time allowed (i) introduction of a loxP site between the right ITR and the E4 promoter for later insertion of a second transcription unit to this vector as described by Suzuki et al. (2015) and (ii) insertion of a rox site into the adjacent BAC-vector region allowing Dre-mediated 3SR for insertion of other CRISPR-Cas components. This resulted in the construct named pBA5-FRT-WH. To bring in the second ACT sequence modified ITR into the construct, we inserted the pO6-A5-WH-CMV-mChe, which carried the left ITR flanked with *ACT sequence* into pBA5-FRT-WH by Flp-mediated 3SR. This insertion delivered the *ACT* adjacent to the left ITR and a mCherry expression cassette in place of the deleted E1 region. Similarly, we constructed the pBWH18/19-C5-mChe in two steps: first modifying the right ITR by recombineering with a synthetic linear DNA fragment containing the additional spacer sequences as shown in Figure 2A (second panel) and then inserting pO6-A5-WH18/19-mChe into pBA5-FRT-WH18/19 by 3SR.

Subsequently, pBWH-C5-mChe-Cas9, pBWH-C5-mChe-gRNA, and pBWH-C5-mChe-DD-Cas9 were constructed by inserting pH-gRNA-Cas9F-iGFP, pH-gRNA-iGFP, or pH-gRNA-DDD-Cas9-iGFP, respectively, by Dre-3SR into pBWH-C5-mChe.

The construct pBAd5-FG40-GFP resembles the pFG40 construct described earlier (Graham, 1984). It was constructed by inserting pO6-A5F-CMV-GFP into pBA5-FRT.

The modified bacmids carrying recombinant HAdV-5 genomes were verified by Sanger sequencing covering the modified positions.

To test CTR on a rAd based on an adenovirus from a different species, we constructed a bacmid coding for HAdV-E4. To generate the HAdV-4-based bacmid, we isolated HAdV-E4 DNA from infected 293A cells as described earlier (Ruzsics et al., 2006). The genomic Ad DNA was assembled according to the approach described by Pan et al. (2018) using the NEBuilder reagents according to the manufacturer’s instruction (New England Biolabs, Frankfurt, Germany) with PCR-amplified vector fragments synthetized on pKSB2 (Ruzsics et al., 2006) template by using primer pairs GHBfor/BWHE04rev and BWHE04for/GHBrev. The primers, which were specific to external vector sequences (BWHE04 for BWHE04rev), were flanked with ACT sequences fused with 5′-proximal 40-bp homologies to the HAdV-E4 genome ends (the oligonucleotide sequences are listed in Supplementary Table 2). NEB 10-beta cells were directly electro-transformed with the dialyzed reaction mixture and selected by 25 μg/ml chloramphenicol. The obtained clones were pre-screened by analysis of their restriction patterns, and three clones, which showed the predicted fragments, were verified by next generation sequencing.

One of these constructs, pBWH-E4, showed 100% homology to the predicted sequence, which was based on the reference sequence for this HAdV-4 strain (GenBank accession no. AY594253) and thus was used in this study as a basis of the species-E-derived rAd. To obtain a more comparable genome size to our HAdV-5 based constructs, we deleted the E3 region between nt 27.002 and nt 31.348 (according to the reference sequence) by recombineering, which was reported not to affect the viability of HAdV-4 (Tian et al., 2019). This construct was coined as pBWH-E4-ΔE3 and was used in this study in the rescue experiments.

The basic features of the rAd-bacmids generated in this study are summarized in Supplementary Table 3. The nucleotide sequences of the basic rAd bacmids tailored for CTR in this study (pBWH-C5-mChe and pBWH-E4-ΔE3) were submitted to GenBank (see Supplementary Table 3 for their accession numbers).

### Rescue of Recombinant Adenoviruses by CRISPR/Cas-Mediated *in vivo* Terminal Resolution

The DNA to be transfected was isolated from bacteria by column purification using the NucleoBond Xtra Midi Kit (Macherey-Nagel, Oensingen, Switzerland) following the manufacturer’s instruction and used directly for transfection of circular constructs. If linearized DNA was transfected for control reasons, 5 μg column-purified bacmid DNA was digested overnight with endonuclease *Pac*I (New England Biolabs, Frankfurt, Germany). The linearized DNA was then extracted with phenol/chloroform and precipitated by ethanol on ice for 1 h. Afterward, the DNA precipitates were collected by centrifugation and washed twice with ethanol (70%, v/v). The pellets were dried and re-suspended in sterile deionized water under sterile conditions. Transfection of 293A and 293A-B2 cells was performed using Lipofectamine 3000 (Thermo Fisher Scientific, Waltham, MA, United States) according to manufacturer’s instructions using 6 μl Lipofectamine and 5 μl P3000 for transfection mixtures, containing 2 μg rAd bacmid and (if applicable) 500 ng helper plasmid DNA, applied to 1 × 10^6^ cells, seeded on one well of a six-well plate 24 h prior to transfection. The transfection mixtures were added directly onto the cell culture media, and the cells were incubated overnight. The next day, the transfected cells were collected by trypsinization, and ∼1.25 × 10^5^ viable cells were seeded to four wells of 24-well plates. The cells were observed daily for focus/plaque formation by fluorescent microscopy for the mCherry-expressing constructs and by phase contrast microscopy for the HAdV-4 based construct, and the foci/plaques were observed and counted daily as soon as the first plaques appeared. If no plaque was observed 14 days after transfection, cultures were declared negative. The final foci/plaque counts were normalized to 1 μg rAd DNA. If required, Shield-1 (TaKaRa, Saint-Germain-en-Laye, France) was added to the cell culture media at the indicated concentrations, starting at the day of transfection until the end of the assay.

### Statistics

Unless stated otherwise, each presented data correspond to three or more biological replicates. The data are represented as mean with standard deviations, unless otherwise specified. Statistical significance was calculated by GraphPad Prism software using unpaired *t*-test with Welch’s correction (Figure 3B), ordinary one-way ANOVA test (Figure 1B), Welch’s ANOVA test (Figures 1A, 2B, 4B) and indicated as follows: ^∗^*p* < 0.05, ^∗∗^*p* < 0.01, ^∗∗∗^*p* < 0.001, ^∗∗∗∗^*p* < 0.0001.

## Results

### Inverted Terminal Repeat-Near CRISPR-Cas-Mediated Cleavage in Cells Yielded Efficient Rescue of Recombinant Adenoviruses Based on HAdV-5

Prior to testing the CRISPR-Cas-driven terminal resolution (CTR), we modified a HAdV-5 (species C) based vector [pBA5-FRT (Ruzsics et al., 2014)] by inserting artificial CRISPR/Cas9 target sequences (ACTs), which reportedly have very low off-target effects to the human genome (Yuen et al., 2017), flanking both ITRs (schematic representation in Figure 1A). ACTs were inserted in two steps: first, the ACT sequence was inserted next to the right ITR of pBA5-FRT, followed by the ACT-modified left ITR along with a marker transcription unit expressing mCherry, resulting in pBWH-C5-mChe. For the expression of Cas9 together with the appropriate sgRNA, which directs the Cas9 nuclease recognizing the entire ACT sequences (sgRNA-Ex, Figure 1A), we constructed a helper plasmid (pAR-gRNA-Cas9F-Amp). As controls, we used (i) the parental vector with the conventionally inserted mCherry expression cassette, rendering it applicable for the standard rescue methodology using *Pac*I linearization *in vitro* (Ruzsics et al., 2014), named pBAd5-mChe, and (ii) the newly constructed pBAd5-FG40-GFP possessing the ITR-fusion as described (Graham, 1984) together with a GFP expression cassette, which could be rescued by transfection of circular DNA directly.

To test CTR and compare it with the commonly used *in vitro* linearization method and one of the methods that are based on transfection of circular DNA, we co-transfected 293A cells with pBWH-C5-mChe and pAR-gRNA-Cas9F-Amp (Figure 1B, CTR 5). As controls, we transfected 293A cells also with linearized pBAd5-mChe (lin5), with circular pBWH-C5-mChe without the helper plasmid (cir5), and circular pBAd5-FG40-GFP, which should be rescued due to its ITR fusion (ITRf5). We observed plaque formation in all settings, except after transfection of circular pBWH-C5-mChe alone, which, as expected, did not yield any detectable infectious particles during the observation period of 14 days post-transfection. Remarkably, the CRISPR/Cas-driven terminal resolution (CTR) yielded a much higher number of plaques (Figure 1B) than any of the positive controls. Moreover, plaque formation was visible already 3–4 days after co-transfection of the CTR components, while the positive controls lin5 (linearized) and ITRf (ITR-fusion), respectively, delivered the first visible foci only at 7–9 days post-transfection. It was previously published that the ITR fusion constructs can be rescued to virus progeny as efficiently from circular DNA as from linear viral DNA (Graham, 1984). Here, we observed a somewhat more efficient rescue using the ITR fusion construct compared to the linearized vector (Figure 1B), supporting the notion that the circular nature of the plasmid contributed to improved rescue efficiency. However, alone, it did not explain the robust increase observed with CTR compared to rescue induced by transfection of the linearized control.

Next, we investigated whether the above observation was indeed due to the Cas9 activity. To this end, the Cas9 protein was fused to a destabilizing domain (DD), thereby achieving conditional Cas9 expression, which was dependent on the presence of the stabilizer molecule Shield-1 (Senturk et al., 2017). This destabilized Cas9 expression cassette was inserted into our CTR-tailored HAdV-5 based bacmid together with the expression cassette for sgRNA-Ex. As control, we constructed a similar bacmid expressing constitutively active Cas9 and sgRNA-Ex as above (pBWH-C5-mChe-DD-Cas9 and pBWH-C5-mChe-Cas9, respectively (see Supplementary Table 3). We transfected 293A cells with these bacmids and treated the pBWH-C5-mChe-DD-Cas9 transfections with different concentrations of Shield-1 and compared the primary rescue efficiency to that of the untreated control construct. As depicted in Figure 1C, the reconstitution efficiency indeed dropped drastically, when Shield-1 was not administered or administered in suboptimal concentration. However, higher concentrations restored the efficiency essentially to the level of the positive control, indicating the critical dependence of the CTR on Cas9 activity.

### Cleavage Sites Located Closer to the Adenovirus Genome Ends Mediated More Efficient Virus Rescue

The CTR using sgRNA-Ex principally may allow rescue of different rAd genomes because the recognition sequence did not overlap with the type-specific ITR sequences. However, this design allowed DNA cleavage not closer than 6–7 base pairs from the rAd genome ends similarly to other methods based on cleavage *in vitro* or in cells (Ghosh-Choudhury et al., 1986; Stanton et al., 2008; Ibanes and Kremer, 2013; Ruzsics et al., 2014). Yet, the targeting sequence of CTR may be designed in a more precise manner, inducing dsDNA breaks exactly at the genome ends. To test this, we designed a new sgRNA (sgRNA-Int5, see Figure 2A first panel), which is supposed to induce cleavage at the genome ends directly at the beginning of HAdV-5 ITRs. Due to the unavoidable overlap with the ITRs, this design should only allow specific cleavage, when the sgRNA fits the adenovirus type that is rescued. To test the efficiency of the CTR after exact cleavage, we co-transfected 293A cells with both CTR-modified HAdV-5 vectors together with either of the two sgRNA expression plasmids coding for sgRNA-Ex or -Int5 in the presence of the Cas9 expression construct pSG5-Cas9F and compared primary rescue efficiencies. As shown in Figure 2B, the sgRNA-Int5-based CTR yielded more than twice as many plaques for the HAdV-C5-based construct than the CTR with sgRNA-Ex, indicating that a proximal cleavage induced more efficient rescue than a distant one. To confirm our conclusion that the distance of the cleavage site from the genome ends matters, we also analyzed the effect of an even more distant cleavage site on CTR. We constructed a rAd5-based construct, which carried the ACT 12 base pairs further away from the ITR ends (see Figure 2A lower panel). On this construct, the sgRNA-Ex should induce Cas9-cleavage 18/19 nucleotide upstream of the ITR ends. Testing the rescue efficiency of this setting revealed a significantly lower recombinant virus rescue compared to each of the other settings, which cut closer (Figure 2B).

### Inverted Terminal Repeat-Near CRISPR-Cas-Mediated Cleavage in Cells Yielded Also Efficient Rescue of Recombinant Adenoviruses Based on HAdV-4

Since the primary rescue efficiency after CTR was improved compared to traditional rescue methods for rAds based on HAdV-5, we wanted to test the efficiency of CTR of rAd derived from another adenovirus species. To this end, a rAd based on HAdV-4 (species E) was constructed from purified HAdV4-DNA by Gibson assembly as described by Pan et al. (2018). However, instead of introducing recognition sequences for restriction endonucleases enabling *in vitro* linearization, we flanked the HAdV-4 genome with *ACT* sequences as done before for the HAdV-5-based constructs (Figure 3A). To allow a better comparison to the HAdV-5-based construct, we deleted the E3 region resulting in the bacmid pBWH-E4-ΔE3. Since our HAdV-4-bacmid carried all essential genes required for adenovirus replication in tissue culture, we could test beside the E1 complementing 293A cells also other permissive cells for supporting CTR. After co-transfection of A549, SKOV-3, and 293A cells with this *ACT*-modified species E bacmid and helper plasmid pAR-gRNA-Cas9F-Amp, which expresses sgRNA-Ex and Cas9, we again observed viral plaque formation in all conditions. However, the plaque formation induced by CTR of the HAdV-E4-based vector was less efficient (Figure 3B) and took also longer (7–9 days) than for the HAdV-C5-based vectors in 293 cells using the general cleavage site (sgRNA-Ex, see Figure 1B). Using A549 or SKOV-3 cells, we could also observe plaque formation with similarly low efficiency, which took 9–14 days.

To test the effect of exact cleavage on the HAdV-4-based vector, we constructed a new helper plasmid, which expressed the sgRNA directing the Cas9 nuclease exactly to the ends of the HAdV-4 genome (pAR-Int4-Cas9F-Amp, see Figure 3A). Again, as for the HAdV-5-based rescues, CTR induced by exact cleavage improved the rescue efficiency drastically (Figure 3B), indicating that the release of the rAd ends with exact cleavage is crucial for efficient rescue.

### Highly Versatile Delivery of CRISPR/Cas Components Allowed Efficient CTR

Next, we explored systematically different methods to provide Cas9 and the sgRNA for CTR. To do this, we compared different delivery modes to provide CRISPR/Cas components for CTR. First, to test stable expression of Cas9, a cell line endogenously expressing Cas9 was generated. After stable transfection of 293A cells with a Flag-tagged Cas9 expression vector (pSG5-Cas9F), individual clones were isolated and examined for Cas9 expression by FACS analysis using anti-Flag-tag primary antibody (Figure 4A). Apart from the control cell line 293A, only anti-Flag signal positive, G418-resistant clones are shown). Additionally, these Flag-specific FACS-positive clones were tested for permissiveness as measured by their ability to reconstitute the CTR-modified HAdV-5-based bacmid pBWH-C5-mChe in the presence of pAR-gRNA-Ex (Figure 4A), which expresses only the sgRNA-Ex. Interestingly, in these assays, we could not find a correlation between Cas9 expression levels and permissiveness for CTR-based rAd rescue. This has already indicated that relatively low levels of Cas9F appear to be sufficient for CTR. However, the assay for CTR in these experiments was not quantitative. Nevertheless, we selected two clones, 293A-Cas9-B2, which expressed Cas9F about threefold higher than others and 293A-Cas9-b5 representing other permissive clones with lower Cas9 expression for further quantitative tests.

Having 293A-Cas9-B2 and b5 established allowed us to compare a comprehensive array of CTR settings representing full plasmid-based approaches and different cell-line-based methods. Using the CTR-modified HAdV-C5-based bacmid as a basis, we merged all essential components of the CTR (the rAd genome, the ACTs, the expression cassettes for both Cas9, and the sgRNA-Ex expression) into a single construct (pBWH-C5-mChe-Cas9) to compare it to CTR, based on helper plasmid co-transfections, as it was shown in the earlier assays in this article. We also tested the newly generated cell line stably expressing Cas9F for rescue of CTR-tailored rAd bacmids in the presence of transient sgRNA expression by a helper plasmid and compared its efficiency to all-in-one-bacmid system, when the bacmid contained the sgRNA expression cassette itself. All approaches yielded similar efficiencies of rAds when we compared the different workflows after transfection of either 293A or 293A-Cas9-B2 or -b5 cells (Figure 4B). These data again indicated that the critical amount of the CRISPR/Cas9-component for CTR could be reached with any basic reconstitution setting.

## Discussion

Adenoviruses represent a well-established viral-vector platform broadly utilized for construction of recombinant vaccines, oncolytic viruses, and gene therapy vehicles. Generation of replication-competent rAd and adenovirus-based first generation vectors is well established. Although the rescue of recombinant viruses and vectors from rAd plasmids is inefficient, for the generation of single rAd constructs or a limited series of them, the standard methodology is evidently sufficient. However, the relative inefficiency of the standard rescue methods prevented the use of this vector platform in many technologies that are based on direct virus rescue upon transfection of genomic plasmids, such as generation of rAd libraries with high diversity or propagation of helper independent high-capacity vectors.

Here, we report a new method for rAd rescue based on CRISPR-Cas-mediated *in vivo* cleavage of circular DNA (CTR) instead of the conventional transfection of *in vitro* linearized DNA. Transfection with circular DNA is several fold more efficient than transfection of linear DNA (Chancham and Hughes, 2001). Accordingly, for adenovirus rescue, the gain of efficiency after transfection of circular DNA in combination with *in vivo* I-SceI cleavage was reported to be about fivefold more efficient than linear DNA-based rescue (Stanton et al., 2008). Thus, the circular transfection alone cannot account for the 30–60-fold increase in rAd rescue by CTR, shown above. To our knowledge, the CTR is the first published approach that allowed cutting out the rAd genome from its circular form precisely at the ends of the wild-type ITRs. Our data show the relevance of this accurate cleavage to free the ITRs, since a more proximal cleavage resulted in higher adenoviral rescue as compared to more distant ones (Figures 2B, 3B). This may explain why the restriction endonuclease-mediated approaches were relatively inefficient, since they always leave extra nucleotides at both ends of the linearized rAd genomes.

The improvement by exact cleavage compared to the near cleavage was remarkably different, when rescue efficiencies of rAds from species C and E were tested. The rescue of the species C construct improved two- to threefold by exact cleavage, while the rescue of the species E construct improved more than sixfold. According to the *in silico* activity scoring (Doench et al., 2014), the sgRNA-Ex (activity score 0.715 and 0.808 targeting E4 and C5, respectively) is far more active than either Int5 or Int4 (0.069 and 0.067, respectively), which definitively does not explain the significant increase in the rescue efficiencies induced by either Int5 or Int4. We believe that this difference is due to the fact that the rAd rescue system that we used here, and which was used in most other cases, was developed for rescuing HAdV-5 vectors after non-exact *in vitro* cleavage reaching optimal results for HAdV-5 with external cleavage, but may not be optimal for HAdV-4-based constructs or for exact cleavages. Notably, the sgRNA-Ex-based CTR functioned less efficiently for the HAdV-4-based construct than the HAdV-5 rescue, yet the exact cleavage induced an increase, which allowed the HAdV-4-based construct to reach almost the level of those of HAdV-5, indicating again the importance of the exact cleavage reaching high efficiency rAd rescue.

Furthermore, the gain of efficiency by the CTR-mediated rAd rescue reported herein may render the rAd vectors possible new candidates for library applications with a potential diversity comparable to lentivirus libraries, which are the standard to date. At the best conditions analyzed here, CTR of rAd5 yielded about 30 plaques/cm^2^ of transfected cell culture. This translates to the generation of libraries with at least 10^4^–10^5^ individual clones, when transfecting 20–30 large cell culture dishes. This is comparable to the diversity and the propagation conditions for optimized lentivirus library productions (McDade et al., 2016). However, there is a considerable difference between the two platforms that relates to the amplification potential of such high content libraries. While the maximal titer for a lentivirus preparation is about ∼10^9^ particles/ml, rAd preparations can reach titers as high as ∼10^13^ particles/ml (Lee et al., 2017). This relatively high amplification potential should allow using library selections for adenovirus vectors based on *in vivo* or *ex vivo* vector applications, which are not standards for lentivirus-vector-based libraries. While it is clear that the appropriate primary efficiency of virus rescue is not the only factor, which determines the overall efficiency and the robustness of library propagations, it probably provides a necessary minimum, which makes the further development of other up- and downstream processes possible.

Furthermore, propagation of high-capacity helper virus-free rAd vectors by plasmid transfection is possible, but the productivity of this approach is extremely low (Lee et al., 2019). To propagate rAd vectors for human gene therapy, this approach would demand an efficiency of at least one order of magnitude higher than the current state of the art. It is possible that its low efficiency is determined by the same factor(s), which we found important for rescuing our first generation vectors in this study. Here, we observed that CTR increased the efficiency of rAd reconstitution by up to 30-fold compared to the conventional method using linear DNA. This was further increased for Ad5-based recombinants about twofold by manipulating the cleavage site of Cas9 to a more proximal position with respect to the genome ends. It would be interesting to test whether this overall ∼50–60-fold increase in efficiency can be transferred to the plasmid-based propagation of high capacity rAds.

Interestingly, when we compared different workflows for rAd rescue (Figure 3B), we did not observe significant differences between the application modes of CTR. These data indicated that the critical amount of the CRISPR/Cas9 component for CTR could be reached in any basic reconstitution setting that we tested, and within this experimental range, their relative abundance was not a significant determinant of rescue efficiency, in contrast to the position of the cleavage sites (see above). However, our quantitative assays measured only primary rescue efficiencies and thus may not account for important differences (for example, in timing or vector yield pro cell, etc.), which may be influenced in experimental virus rescue applications where plaque formation does not need to be recorded, such as library rescue or plasmid-based rescue of replication incompetent high-capacity vectors.

## Data Availability Statement

The datasets presented in this study can be found in online repositories. The names of the repository/repositories and accession number(s) can be found below: https://www.ncbi.nlm.nih.gov/genbank/, MW775630, MW775628, MW775629, MW775625, MW775626, MW775624, OK268108, MW775631, MW775632, MW775633, and OK268109.

## Author Contributions

AR and ZR conceived the project. AR, H-GB, and ZR designed the experiments and analysis. AR, JF, and ZR performed the experiments and analyzed the data. AR, JF, H-GB, and ZR wrote and reviewed the manuscript. All authors contributed to the article and approved the submitted version.

## Conflict of Interest

AR, JF, H-GB, and ZR are co-inventors in patent application EP20198944 by the Albert Ludwig University of Freiburg that describes the use of CRISPR/Cas-mediated rescue of recombinant adenoviruses from circular plasmids.

## Publisher’s Note

All claims expressed in this article are solely those of the authors and do not necessarily represent those of their affiliated organizations, or those of the publisher, the editors and the reviewers. Any product that may be evaluated in this article, or claim that may be made by its manufacturer, is not guaranteed or endorsed by the publisher.
