# Supplementary material for: Rescue of Recombinant Adenoviruses by CRISPR/Cas-Mediated in vivo Terminal Resolution
Source: Front Microbiol. 2022 Mar 18;13:854690. doi: 10.3389/fmicb.2022.854690 (PMC8975557; doi:10.3389/fmicb.2022.854690)
Supplement: Supplementary file 1 [file Data_Sheet_1.PDF]

Supplementary Material

[André Riedl, Julian Fischer, Hans-Gerhard Burgert, Zsolt Ruzsics
Rescue of recombinant adenoviruses by CRISPR/Cas-mediated *in vivo* terminal resolution]

Supplementary Table 1

Plasmid constructs used in this study

**Plasmids**		
**Name**	**Properties**	**GenBank**
pH-gRNA-iGFP	Constitutive gRNA-Ex expression by U6-promoter, targeting *warhead* sequence; single rox site for Dre-recombination; Constitutive SpCas9-Flag-NLS expression under EF1α promoter; ampicillin resistance; conditional ori*	MW775630
pH-gRNA-Cas9F-iGFP	Constitutive gRNA-Ex expression by U6-promoter, targeting *warhead* sequence; single rox site for Dre-recombination; Constitutive SpCas9-Flag-NLS expression under EF1α promoter; internal ribosomal entry site (IRES) driven GFP downstream of SpCas9; ampicillin resistance; conditional ori*	MW775628
pH-gRNA-DDD-iGFP	Constitutive -gRNA-Ex expression by U6-promoter, targeting *warhead* sequence; single rox site for Dre-recombination; Constitutive expression of destabilized (DD) SpCas9-Flag-NLS under EF1α promoter; IRES driven GFP downstream of SpCas9; ampicillin resistance; conditional ori*	MW775629
pAR-gRNA-Ex	Constitutive gRNA-Ex expression under human U6 promoter, targeting *ACT* sequence; ampicillin resistance	MW775625
pAR-gRNA-Int5	Constitutive gRNA-Int5 expression by U6 promoter, targeting HAdC5-ITR sequence; ampicillin resistance	MW775626
pAR-gRNA-Cas9F-Amp	Constitutive gRNA-Ex expression by U6-promoter, targeting *ACT* sequence; Constitutive SpCas9-Flag-NLS under EF1α promoter; ampicillin resistance	MW775624
pAR-Int4-Cas9F-Amp	Constitutive gRNA-Int4 expression by U6-promoter, targeting HAdD4-ITR sequence; Constitutive SpCas9-Flag-NLS under EF1α promoter; ampicillin resistance	OK268108
pO6-A5-WH-CMV-mChe	Constitutive mCherry expression by HCMV-ie-promoter; upstream of L-ITR (HAdV-C5) *warhead* sequence; kanamycin resistance; ori6Kγ ori	MW775631
pSG5-Cas9F	Expression of SpCas9-Flag-NLS under SV40 promoter; primary transcript with beta-globin intron; ampicillin resistance	MW775632

*Hashimoto-Gotoh, T., Franklin, F. C., Nordheim, A. & Timmis, K. N. Specific-purpose plasmid cloning vectors. I. Low copy number, temperature-sensitive, mobilization-defective pSC101-derived containment vectors. *Gene* **16**, 227-235, doi:10.1016/0378-1119(81)90079-2 (1981).

Supplementary Table 2

Oligonucleotides used in this study

**Oligonucleotides**	
**Name**	**Sequence (5‘- 3‘)**
GHBfor	CCGCGTGTGTACCTCTACCTGGAGTTTTTCCCACGGTGGA
BWHE04rev	TTAACTCACACAAAAAAAAATAAGGTATATTATATAGATAGCCTCCGG GGTCCACTGCAATTATAAACTCGACAGCGACACACTTGC
BWHE04for	TTAACTCACACAAAAAAAAATAAGGTATATTATATAGATAGCCTCCGG GGTCCACTGCAATTACTTCTCGACCAATTCTCATGTTTGAC
GHBrev	TCCACCGTGGGAAAAACTCCAGGTAGAGGTACACACGCGG

Supplementary Table 3

Recombinant adenovirus BACs		
**Name**	**Properties**	**GenBank**
pBAd5-FG40-GFP	Bacmid carrying HAdV-C5-ΔE1ΔE3 genome; ITR-fusion described for pFG40 in the place of the left ITR*; expression of GFP under HCMV-ie-promoter; chloramphenicol and kanamycin resistance	N.A.[+]
pBWH-C5-mChe	Bacmid carrying HAdV-C5-ΔE1ΔE3 genome; both ITRs flanked by *warhead* sequences; expression of mCherry under HCMV-ie-promoter; chloramphenicol and kanamycin resistance	MW775633
pBWH-C5-mChe-Cas9	Bacmid based on pBWH-C5-mChe which was modified by insertion of pH-gRNA-Cas9F-iGFP, which carried an expression cassette of gRNA-Ex under the control of U6-promoter and an expression cassette of SpCas9-Flag-NLS under EF1α-promoter; chloramphenicol, kanamycin, and ampicillin resistance	N.A.
pBWH-C5-mChe-DD-Cas9	Bacmid based on pBWH-C5-mChe which was modified by insertion of pH-gRNA-DDD-iGFP, which carried an expression cassette of gRNA-Ex under the control of U6-promoter and an expression cassette of destabilized SpCas9-Flag-NLS under EF1α-promoter; chloramphenicol, kanamycin, and ampicillin resistance	N.A.
pBWH-C5-mChe-Cas9	Bacmid based on pBWH-C5-mChe which was modified by insertion of pH-gRNA-DDD-iGFP, which carried an expression cassette of gRNA-Ex under the control of U6-promoter and an expression cassette of destabilized SpCas9-Flag-NLS under EF1α-promoter; chloramphenicol, kanamycin, and ampicillin resistance	N.A.
pBWH-C5-mChe-gRNA-Cas9	Bacmid based on pBWH-C5-mChe which was modified by insertion of pH-gRNA-DDD-iGFP, which carried an expression cassette of gRNA-Ex under the control of U6-promoter and an expression cassette of destabilized SpCas9-Flag-NLS under EF1α-promoter; chloramphenicol, kanamycin, and ampicillin resistance	N.A.
pBWH-E4-DE3	Bacmid carrying E3 deleted HAdV-4 genome cloned into pKSB2; both ITRs flanked by *ACT* sequences; chloramphenicol resistance	OK268109

*Graham, F. L. Covalently closed circles of human adenovirus DNA are infectious. *The EMBO journal* **3**, 2917-2922 (1984).

[+]not applicable
